# Synthesis of novel conjugates of a saccharide, amino acids, nucleobase and the evaluation of their cell compatibility

**DOI:** 10.3762/bjoc.10.250

**Published:** 2014-10-16

**Authors:** Dan Yuan, Xuewen Du, Junfeng Shi, Ning Zhou, Abdulgader Ahmed Baoum, Bing Xu

**Affiliations:** 1 Department of Chemistry, Brandeis University, 415 South Street, MS015, Waltham, MA 02453, USA; 2 Department of Chemistry, King Abdulaziz University, Jeddah, Saudi Arabia

**Keywords:** cell compatibility, nucleobase, peptides, saccharide

## Abstract

This article reports the synthesis of a novel type of conjugate of three fundamental biological build blocks (i.e., saccharide, amino acids, and nucleobase) and their cell compatibility. The facile synthesis starts with the synthesis of nucleobase and saccharide derivatives, then uses solid-phase peptide synthesis (SPPS) to build the peptide segment (Phe-Arg-Gly-Asp or naphthAla-Phe-Arg-Gly-Asp with fully protected groups), and later, an amidation reaction in liquid phase connects these three parts together. The overall yield of these multiple step synthesis is about 34%. Besides exhibiting excellent solubility, these conjugates of saccharide–amino acids–nucleobase (SAN), like the previously reported conjugates of nucleobase–amino acids–saccharide (NAS) and nucleobase–saccharide–amino acids (NSA), are mammalian cell compatible.

## Introduction

As a result of evolution, nature selects saccharides, peptides, and nucleobases as the fundamental building blocks for the creation of biomacromolecules, which lay the molecular foundations of life. This simple fact and the self-assembly of small molecules in water have inspired us to explore the conjugates of those three types of building blocks for generating supramolecular nanofibers in water [1–15]. Recently, we have demonstrated that not only the conjugates of nucleobase–amino acids–saccharide (NAS) [16–18] but also the conjugates of nucleobase–saccharide–amino acids (NSA) [19] are able to self-assemble to form supramolecular hydrogels and exhibit promising biological functions, such as promoting the development of zygotes [20]. Moreover, both NAS- and NSA-type conjugates exhibit excellent cell compatibility. Except for a few known motifs (naphthalene [21–22], Fmoc [23–24], lipid [25]), it is still challenging to judge which molecules could self-assemble to form ordered nanostructures. This challenge requires the molecular engineering of the conjugates and intense study of their properties to provide a molecular basis for the understanding of the fundamental correlation between molecular structure and self-assembly.

**Figure 1:**
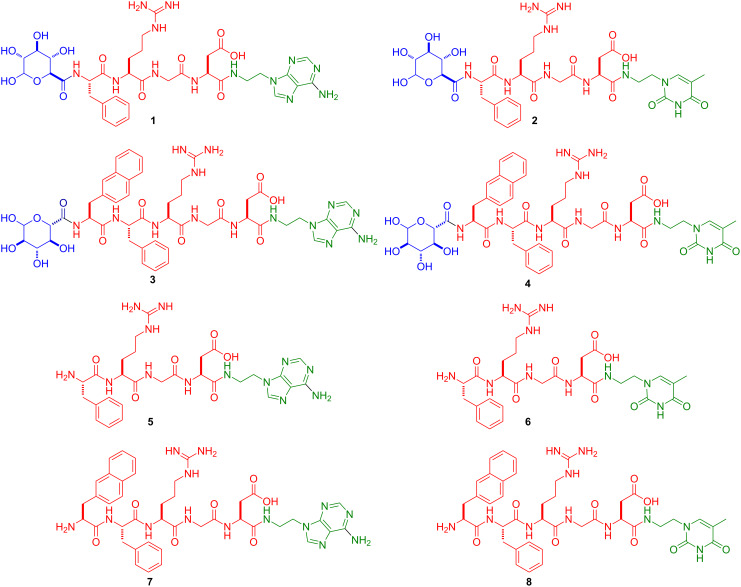
Chemical structures of the saccharide–amino acids–nucleobase conjugates (SAN, **1**–**4**) and amino acids–nucleobase (AN, **5**–**8**).

Here we investigate new molecular conjugates made of basic building blocks by connecting a saccharide segment to amino acids, and nucleobase (i.e., saccharide–amino acids–nucleobase, we term it SAN-type). As shown in Figure 1, in the conjugates **1**–**4**, the saccharide is glucuronic acid, the amino acid segment consists of Phe-Arg-Gly-Asp or naphthAla (3-(2-naphthyl)-alanine)-Phe-Arg-Gly-Asp, and the nucleobase is thymine or adenine. Our results show that the conjugates synthesized and examined in this work exhibit excellent cell compatibility but are unable to self-assemble in water to form hydrogels. Their excellent solubility in water implies that these molecules may find applications for breaking up molecular aggregates in water, a much-needed property for warranting genuine monomeric ligand–receptor interactions. The conjugates **5**–**8**, without a saccharide segment, have good solubility at acidic conditions, also present great cell compatibility and dissolve well in water. These results imply that glucuronic acid is unlikely to be the key factor for cell compatibility of SAN-type conjugates.

## Results and Discussion

### Molecular design

Figure 1 shows the structures of the designed molecules based on the mutation of the sequence of the connection of nucleobase, amino acids, and saccharide. To explore the properties of the conjugates dependent on the relative positions of the building blocks we synthesized conjugates having the following order: saccharide–amino acids–nucleobase (i.e., SAN-type, **1**–**4**). Although oligosaccharide moieties serve as constituents of glycoproteins in a broad range of cell–cell and cell–matrix recognition events, the introduction of glucuronic acid into peptides at the *N*-terminus (Figure 1) is rare and worth the exploration [26]. As an amino acid, Phe or naphthAla increases molecular aromatic–aromatic interactions [19,21,27]. Arg-Gly-Asp, which is a well-established tripeptidic epitope, that modulates mammalian cell adhesion through binding with integrins on the cell membrane [28–29]. Thymine or adenine, as a unique types of heteroatom aromatics, not only promote self-assembly [30], but also have the capability for DNA delivery [16]. To examine the role of the saccharide, we also designed and synthesized the respective conjugates of amino acids and nucleobase without the glucuronic moiety (**5**–**8**) for comparison.

### Synthesis

**Scheme 1:**
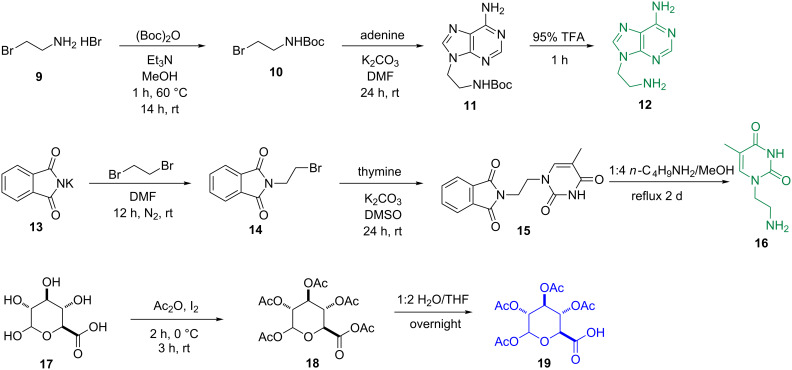
Synthesis of nucleobase (thymine and adenine) and saccharide (glucuronic acid) derivatives **12**, **16**, and **19**.

**Scheme 2:**
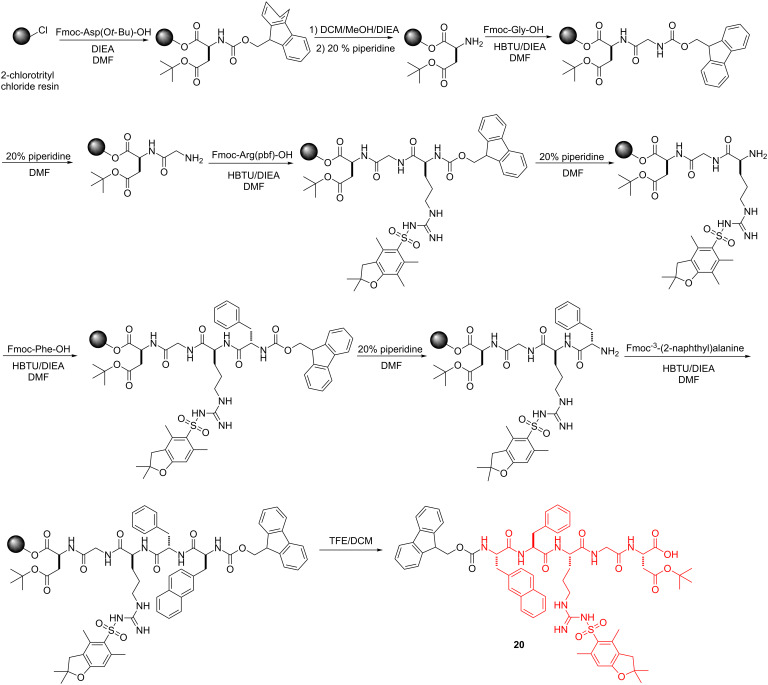
Solid-phase peptide synthesis of peptide segment Fmoc-naphthAla-Phe-Arg(pbf)-Gly-Asn(O*t*-Bu)-OH (**20**).

**Scheme 3:**
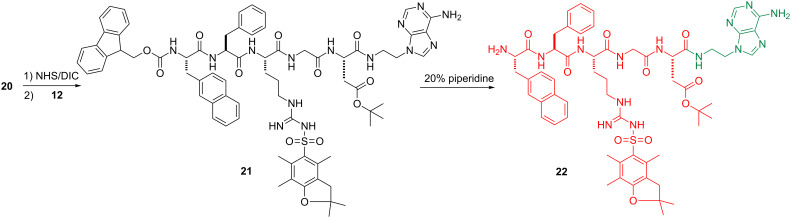
Synthesis of H-naphthAla-Phe-Arg(pbf)-Gly-Asn(O*t*-Bu)-adenine (**22**).

**Scheme 4:**
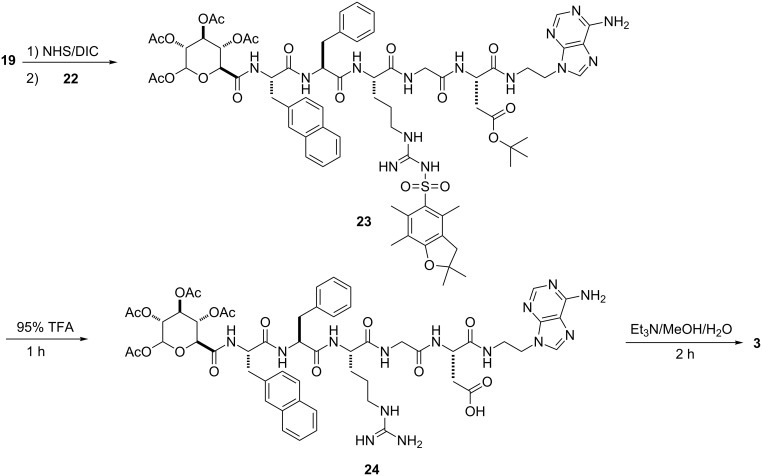
Synthesis of saccharide–amino acids–nucleobase conjugate **3**.

Schemes 1–4 show the syntheses of the SAN conjugates formed by the reaction of the amino acid segment with the nucleobase and the saccharide derivative. The key steps include *N*-alkylation, acetylation, solid-phase peptide synthesis (SPPS) and *N*-hydroxysuccinimide (NHS)/*N*,*N*-diisopropylcarbodiimide (DIC)-catalyzed amidation reaction. As demonstrated by the example of the synthesis of **3**, the use of reported methods [31–35] (Scheme 1) affords the nucleobase and saccharide derivatives **12**, **16**, and **19**. We used SPPS [36] (Scheme 2) to synthesize the fully protected naphthAla-Phe-Arg-Gly-Asp (**20**). After loading the first amino acid (Fmoc-Asp(O*t*-Bu)-OH) on the 2-chlorotrityl chloride resin, we blocked the resin by dichloromethane (DCM)/methanol (MeOH)/*N,N*-diisopropylethylamine (DIEA) (8:1.5:0.5), next removed the Fmoc protecting group by 20% piperidine in *N*,*N*-dimethylformamide (DMF), then used the coupling reagent *N,N,N’,N’*-tetramethyl-*O*-(1*H*-benzotriazol-1-yl)uronium hexafluorophosphate (HBTU)/DIEA to connect the following *N*-Fmoc amino acid (i.e., Fmoc-Gly-OH, Fmoc-Arg(pbf)-OH, Fmoc-Phe-OH, Fmoc-3-(2-naphthyl)alanine) by repeating the deprotection and coupling steps. At last, we cleaved the pentapeptide **20** with all protected groups from the resin by using trifluoroethanol (TFE)/DCM (2/8, 2 × 1 hour). The reaction of **20** and NHS/DIC activates the carboxyl group on **20** to couple with **12** at pH 8.5, which results in **21**. The subsequent removal of the Fmoc group provides the protected nucleopeptide **22** [30] (Scheme 3). The activation of **19** to react with **22** and the subsequent removal of Fmoc results in **23**. Finally, treatment of **23** with 95% trifluoroacetic acid (TFA) for 1 hour and triethylamine (Et_3_N)/MeOH/H_2_O 1:4:5) for 2 hours affords conjugate **3** (Scheme 4). By changing the nucleobase and using the similar reaction procedures, we obtained the rest of the conjugates shown in Figure 1.

### Inability of hydrogelation

Unlike the NAS- and NSA-type conjugates, none of the SAN-type conjugates form hydrogels at the conditions tested. As shown in Supporting Information File 1, Figure S1A, B, **1** or **2** form a viscous aqueous solution at a concentration of 3 wt % (pH 4.0) after one week aging. Although our previous study shows that the incorporation of naphthAla, a naphthalene-containing unnatural amino acid, into NSA is able to induce hydrogelation [19], the introduction of naphthAla to **1** or **2**, which makes conjugate **3** or **4**, however, is unable to result in hydrogelation (Supporting Information File 1, Figure S2A and S2B). The corresponding TEM images showed that conjugates **1**–**4** all form aggregates (upon drying) without ordered structures. Meanwhile, the removal of the saccharide from the SAN-type conjugates, which forms compound **5–8**, results in no hydrogel (Supporting Information File 1, Figure S1C, D and Figure S2C, D). These results suggest that the sequences of the three types of fundamental biological building blocks in the conjugates are critical for their self-assembly.

#### Cell compatibility

**Figure 2:**
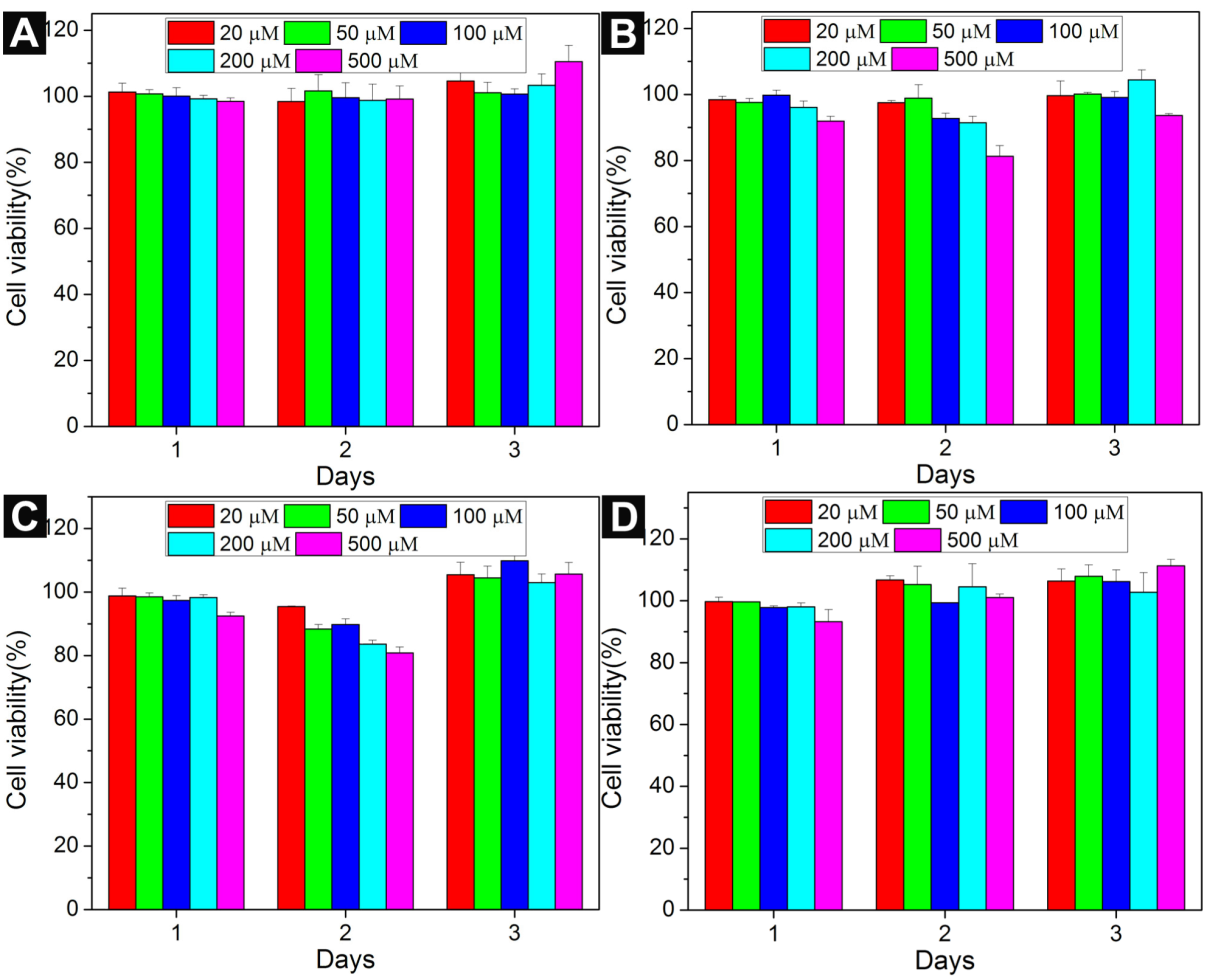
Cell viability of HeLa cells incubated with (A) **1**, (B) **2**, (c) **5**, (D) **6** at different concentrations for 3 days.

**Figure 3:**
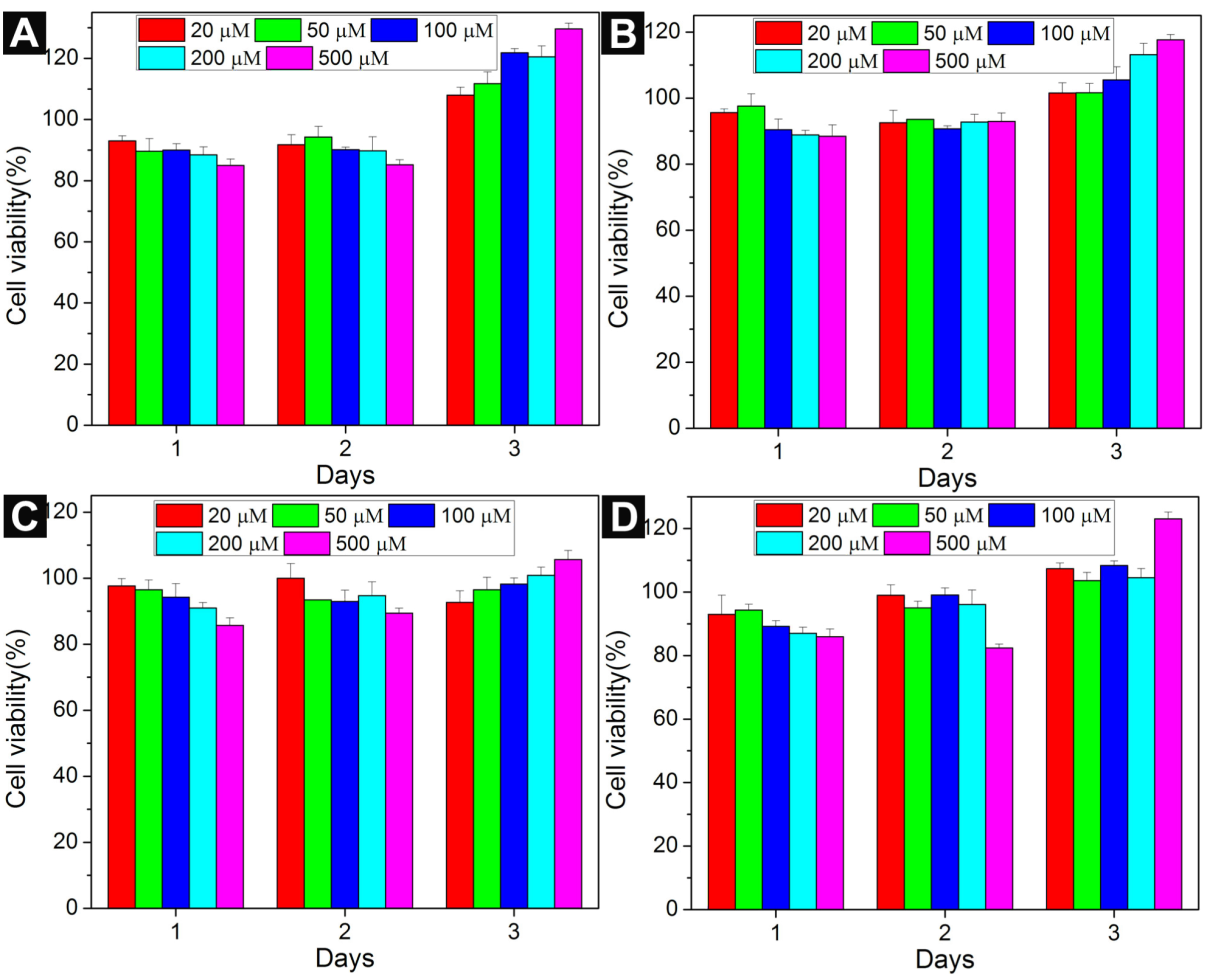
Cell viability of HeLa cells incubated with (A) **3**, (B) **4**, (C) **7**, (D) **8** at different concentrations for 3 days.

To investigate the cell compatibility of the conjugates, we incubated compounds **1**–**8** with HeLa cells [37] for 3 days at a concentration range from 20 μM to 500 μM. We compared the cytotoxicity of conjugates **1**, **2**, **5**, and **6** in Figure 2. Compounds **5** and **6** serve as the control for compounds **1** and **2** since they do not have a saccharide part. The viability of HeLa cells incubated with **1**, **2**, **5**, and **6** is around 100% at the study concentrations for 3 days. Also conjugates **3**, **4**, **7**, and **8** exhibit little toxicity to HeLa cells (Figure 3). Particularly, the cytotoxicities of conjugates **1**, **3**–**5**, and **8** are slightly higher than 100% at 72 h, it is likely originate from the metabolic activity increase in the HeLa cells treated by **1**, **3–5**, and **8** [38]. These results are consistent with our previous reports that NAS- and NSA-type conjugates are cell compatible, indicating that the sequences of the three types of building blocks have slight influences to their cell responses. Based on these results, we speculate that the cell compatibility of this type of conjugate is not only due to the natural building blocks, but also may originate from their inability to form aggregates at low concentrations.

## Conclusion

In summary, this article reports the synthesis of novel conjugates containing three fundamental biological build blocks (saccharide, amino acids, and nucleobase) and their cell compatibility. The attachment of the saccharide to the *N*-terminal of the peptide or the nucleobase to the *C*-terminal of the peptide apparently reduces the self-assembly ability of these conjugates in water. Although the atomistic details remain to be elucidated, the observation itself is intriguing. These results provide a new understanding of the self-assembly of the conjugates consisting of fundamental building blocks of biomacromolecules. One potential application of these SAN-type conjugates is to minimize unwanted aggregates of drug candidates for increasing the efficacy of drugs, which is a direction worth exploring.

## Supporting Information

File 1 General section, experimental section, TEM images of solutions of **1**–**8**.
