# Supplementary material for: Synthesis of novel conjugates of a saccharide, amino acids, nucleobase and the evaluation of their cell compatibility
Source: Beilstein J Org Chem. 2014 Oct 16;10:2406–13. doi: 10.3762/bjoc.10.250 (PMC4222440; doi:10.3762/bjoc.10.250)
Supplement: File 1 — General section, experimental section, TEM images of solutions of 1–8. [file Beilstein_J_Org_Chem-10-2406-s001.pdf]

Supporting Information

for

Synthesis of novel conjugates of a saccharide, amino acids, nucleobase and the evaluation of their cell compatibility

Dan Yuan[1], Xuewen Du[1], Junfeng Shi[1], Ning zhou[1], Abdulgader Ahmed Baoum,[2] and Bing Xu*[1]

Address: [1]Department of Chemistry, Brandeis University, 415 South Street, MS015, Waltham, MA 02453, USA. [2]Department of Chemistry, King Abdulaziz University, Jeddah, Saudi Arabia.

Email: Bing Xu - bxu@brandeis.edu

* Corresponding author

General section, experimental section, TEM images of solutions of 1–8.

1. General

1.1 Materials

All of the solvents and chemical reagents were used as received from the commercial sources without further purification unless otherwise noted. Amino acids and HBTU were purchased form GL Biochem (Shanghai) Ltd. D-glucuronic acid was

purchased from Alfa-Aesar. Thymine, Adenine, *N*-hydroxysuccinimide (NHS), *N*,*N*-diisopropylcarbodiimide (DIC), Di-tert-butyl dicarbonate (Boc_2O) were purchased from Acros Organics. 2-Bromoethylamine hydrobromide was purchased from TCI America. Other materials and solvents were purchased form Fisher Scientific. The HeLa cell line (CCL2) was purchased from American Type Culture Collection. All of the media were purchased from Invitrogen.

1.2 Spectrometers:

^{1}H NMR was run on Varian Unity Inova 400. LC-MS was taken on Waters Acquity ultra Performance LC with Waters MICROMASS detector. TEM images were collected on Morgagni 268 transmission electron microscope. MTT assay for cell viability test was obtained on DTX880 Multimode Detector.

2. Experimental

Compounds **10**-**19** were prepared according to literatures [1-5].

Syntheis of 10: 2-Bromoethylamine hydrobromide (2.0 g, 9.8 mmol) was dissolved in methanol (40 mL) and to it di-*tert*-butyl dicarbonate (4.28g, 19.6 mmol) along with triethylamine (1.4 mL, 10 mmol) was added. The mixture solution was stirred at 60 ^oC for 1 h then at room temperature for 14 h. The resulting solution was evaporated under reduced pressure, and dichloromethane (150 ml) was added, then washed with 1M HCl (4 x 50 mL) and saturated aqueous NaCl (3 x 50 mL). The organic phase was dried over Na_2SO_4, filtered, and concentrated under reduced pressure, and oil was

obtained (1.88g, 85.7 %). ^{1}H NMR (400 MHz, $CDCl_3$) δ: 4.94 (br. s, 1H, N*H*), 3.53 (t, 2H, C*H*$_2$NH), 3.46 (t, 2H, BrC*H*$_2$), 1.45 (s, 9H, Boc).

Synthesis of 11: Compound **10** (1.88 g, 8.4 mmol) was added to DMF solution (50 mL) of adenine (1.14 g, 8.4 mmol) with K_2CO_3 (2.32 g, 16.8 mmol), and the solution was stirred at room temperature for 24 h. After removing the solvent by rotary evaporation, compound thus obtained was purified by flash chromatography on silica gel. The solvent was removed under reduced pressure to obtain a white powder (1.6 g, 68.5 %). ^{1}H NMR (400 MHz, DMSO-d_6) δ: 8.12 (s, 1H, adenine H), 7.99 (s, 1H, adenine H), 7.14 (s, 2H, N*H*$_2$), 6.96 (t, 1H, N*H*), 4.17 (t, 2H, NC*H*$_2$), 3.35 (m, 2H, C*H*$_2$NH), 1.31 (s, 9H, Boc).

Synthesis of 12: Compound **11** (1.6 g, 5.75 mmol) was dissolved in 10 mL TFA/H_2O (95: 5) and stirred at room temperature for 1 h. The solvent was removed by air-blowing, and diethyl ether was added. The precipitate formed was filtered off under reduced pressure to get compound **12** (0.84 g, 82.1%). ^{1}H NMR (400 MHz, DMSO-d_6) δ: 8.33 (s, 1H, adenine H), 8.26 (s, 1H, adenine H), 7.92 (s, 2H, N*H*$_2$), 4.43 (t, 2H, NC*H*$_2$), 3.34 (m, 2H, C*H*$_2$NH$_2$).

Syntheis of 14: To a well-stirred solution of potassium phthalimide **13** (5.00 g, 27.0 mmol) in DMF (10 ml) was added 1, 2-dibromoethane (6.91 ml, 81.0 mmol) and the reaction mixture was stirred 15 h under nitrogen. The solvent was removed under

reduced pressure and the residue was dissolved in ethyl acetate (200 ml) and extracted with water (2 × 100 ml). The organic layer was washed successively with saturated aqueous NH_4Cl and NaCl, dried over Na_2SO_4, filtered, and concentrated under reduced pressure to afford **14** (6.15 g, 89.6 %) as a white crystalline solid. ^{1}H NMR (400 MHz, $CDCl_3$) δ: 7.85 (m, 2H, ArH), 7.72 (m, 2H, ArH), 4.10 (t, 2H, NCH_2), 3.58 (t, 2H, CH_2Br).

Syntheis of 15: Thymine (2.50 g, 19.8 mmol) was dissolved in DMSO (50 ml) and then treated with K_2CO_3 (2.76 g, 20.0 mmol) and **14** (2.60 g, 10.3 mmol) for 12 h at room temperature. After the precipitate had been filtered off, the filtrate was concentrated under reduced pressure to produce a viscous yellowish liquid. The liquid was diluted with dichloromethane (50 ml) and extracted with water (25 ml). The water layer was extracted further with dichloromethane (2 x 25 ml). The combined organic layers were concentrated under reduced pressure and the resulting oil was dissolved in a small volume of dichloromethane and induced to crystallize with hexane to obtain **15** (3.12 g, 52.7 %) as a white powder. ^{1}H NMR (400 MHz, DMSO-d_6) δ: 7.83-7.78 (m, 4H, ArH), 7.47 (s, 1H, thymine 6-H), 3.96 (t, 2H, NCH_2CH_2N), 3.80 (t, 2H, NCH_2CH_2N), 1.61 (s, 3H, CH_3).

Syntheis of 16: Compound **15** (1.50 g, 5.0 mmol) was dissolved in n-butylamine/methanol (1: 4, 30 mL) and the solution was stirred under reflux for 2 days. The reaction mixture was concentrated under reduced pressure and then

dissolved in 0.5 M HCl (75 ml). After extraction with diethyl ether (75 ml), the aqueous phase was evaporated under reduced pressure. The residue was dissolved in benzene/methanol (1: 1, 32 mL) to form an azeotropic mixture, which was evaporated under reduced pressure. A solid mass was formed, which was recrystallized from a mixture of MeOH/diethylether/chloroform (1:1:1) to obtain **16** (600 mg, 70.9 %) as a white powder. ^{1}H NMR (400 MHz, DMSO-d_6) δ: 7.40 (s, 1H, thymine 6-H), 3.87 (t, 2H, NCH_2), 3.07 (br. s, 2H, CH_2NH$_2$), 1.71 (s, 3H, CH_3).

Synthesis of 18: To a well stirred, 0 ^{o}C solution of **17** (1 g, 5.15 mmol) in acetic anhydride (15 mL) was added slowly iodine (70 mg, 0.28 mmol). The red solution was stirred for 2 h on ice and for further 3 h at room temperature. Acetic anhydride was mostly removed under reduced pressure and the remaining mixture was dissolved in dichloromethane (12 mL). The organic layer was then washed with $Na_2S_2O_3$ (1.0 M, 2 x 16 mL), dried ($MgSO_4$), filtered, and concentrated to afford **18** as a white powder. The β-anomer can be separated from its α-anomer (present as minor impurity) by recrystallization from dichloromethane/petro-leum ether (2/3) (1.58 g, 75.9 %). ^{1}H NMR (400 MHz, $CDCl_3$) δ: 5.76 (d, 1H, H-1), 5.31 (t, 1H, H-4), 5.22 (t, 1H, H-3), 5.05 (t, 1H, H-2), 4.26 (d, 1H, H-5), 2.21 (s, 3H, COOCOCH_3), 2.01, 1.99, 1.98, 1.97 (each s, each 3H, each COCH_3).

Synthesis of 19: Compound **18** (1.38 g, 3.42 mmol) was dissolved in water and THF (1: 2, 30 mL) and stirred overnight at room temperature. The solution was

concentrated and the product extracted into dichloromethane (3 x 50 mL), dried over $MgSO_4$, and filtered. After evaporation under reduced pressure, a white fluffy material was obtained and identified as **19** (1.14 g, 92.1 %). ^{1}H NMR (400 MHz, $CDCl_3$) δ: 5.76 (d, 1H, H-1), 5.29 (m, 2H, H-3 and H-4 overlapping), 5.11 (t, 1H, H-2), 4.20 (m, 1H, H-5), 3.57 (br. s, 1H, COO*H*), 2.17, 2.10, 2.02, 2.01 (each s, each 3H, each $COCH_3$).

Synthesis of 20: compound **20** was obtained by Solid Phase Peptide Synthesis (SPPS) [6]. All processes were carried out under N_2 environment. ^{1}H NMR (400 MHz, DMSO-d_6) δ: 8.19 (m, 3H, N*H*), 8.05 (s, 1H, N*H*), 7.83 (d, 3H, ArH), 7.77 (m, 3H, ArH), 7.63 (d, 1H, ArH), 7.55 (m, 2H, ArH), 7.47 (m, 3H, ArH), 7.36 (q, 2H, ArH), 7.24 (m, 5H, ArH), 7.14 (q, 2H, ArH), 6.67, 6.42 (each br. s, 2H, N*H*CN*H*), 4.58 (m, 1H, C*H*CH_2CO), 4.32 (m, 2H, C*H*CH_2C), 4.07 (m, 3H, CHCH_2O and C*H*CH_2CH_2 overlapping), 3.89 (t, 1H, C*H*CH_2O), 3.75 (d, 2H, NHCH_2CO), 3.09 (m, 4H, CHCH_2C), 2.90 (m, 4H, CHCH_2CO and CCH_2C overlapping), 2.65 (m, 2H, CH_2CH_2NH), 2.47, 2,41, 1.97 (each s, each 3H, each CCH_3), 1.69 (br. s, 1H, CN*H*), 1.45 (m, 4H, CCH_2CH_2), 1.37, 1.34 (each s, 15H, $C(CH_3)_3$ and $C(CH_3)_2$).

Synthesis of 21: DIC (536 μL, 3.43 mmol) was added to tetrahydrofuran (20 mL) solution of **20** (1.2g, 0.98 mmol) and NHS (395.4 mg, 3.43 mmol) and stirred at room temperature for 3 h. Then the resulting solution was added dropwise to a well stirred water solution (5 mL) of compound **12** (436 mg, 2.45 mmol) which the pH was adjusted to 8.5 by sodium carbonate. The mixture was stirred overnight at room

temperature. The solvent was evaporated under reduced pressure. The residue was washed by water, filtered, and purified by flash chromatography on silica gel to yield **21** (712 mg, 52.6 %) as a white powder. ^{1}H NMR (400 MHz, DMSO-d_6) δ: 8.21 (m, 3H, N*H*), 8.16 (m, 3H, N*H* and adenine H overlapping) 7.99 (s, 1H, adenine H), 7.83 (m, 3H, ArH), 7.76 (m, 3H, ArH), 7.62 (d, 1H, ArH), 7.55 (m, 2H, ArH), 7.45 (m, 3H, ArH), 7.36 (q, 2H, ArH), 7.21 (m, 7H, ArH), 6.65, 6.42 (each br. s, 2H, N*H*CN*H*), 4.59 (m, 1H, C*H*CH_2CO), 4.32 (m, 2H, C*H*CH_2C), 4.17 (t, 2H, $NHCH_2CH_2N$), 4.07 (m, 2H, $CHCH_2O$ and C*H*CH_2CH_2 overlapping), 3.88 (s, 1H, C*H*CH_2O), 3.74 (s, 2H, $NHCH_2CO$), 3.43 (m, 2H, $NHCH_2CH_2N$), 3.08 (m, 4H, $CHCH_2C$), 2.89 (m, 4H, $CHCH_2CO$ and CCH_2C overlapping), 2.62 (m, 2H, CH_2CH_2NH), 2.46, 2,40, 1.97 (each s, each 3H, each CCH_3), 1.69 (br. s, 1H, CN*H*), 1.44 (m, 4H, CCH_2CH_2), 1.36, 1.34 (each s, 15H, $C(CH_3)_3$ and $C(CH_3)_2$). LCMS (ESI) (m/z): C73H84N14O12S, calcd 1380.61; found 1379.97 $[M-1]^-$, 1381.92 $[M+1]^+$.

Synthesis of 22: Compound **21** (557 mg, 0.40 mmol) was treated with 20% piperidine in chloroform for 30 min. The solution was concentrated under reduced pressure and the product **22** (401 mg, 86.5 %) was obtained after purification by flash chromatography on silica gel. ^{1}H NMR (400 MHz, DMSO-d_6) δ: 8.29 (d, 1H, N*H*), 8.14 (m, 5H, N*H* and adenine H overlapping), 8.00 (s, 1H, adenine H), 7.79 (m, 3H, ArH), 7.65 (s, 1H, ArH), 7.44 (m, 2H, ArH), 7.31 (d, 1H, ArH), 7.16 (m, 5H, ArH), 6.75, 6.43 (each br. s, 2H, N*H*CN*H*), 4.60 (m, 1H, C*H*CH_2CO), 4.51 (m, 1H, NH_2CHCO), 4.27 (m, 2H, C*H*CH_2C and C*H*CH_2CH_2 overlapping), 4.17 (t, 2H, $NHCH_2CH_2N$), 3.74 (t, 2H,

NHCH_2CO), 3.45 (m, 2H, NHCH_2CH$_2$N), 3.03 (m, 4H, C*H*CH$_2$C), 2.88 (m, 4H, CHCH_2CO and CCH_2C overlapping), 2.61 (m, 2H, CH$_2$CH_2NH), 2.46, 2.41, 1.98 (each s, each 3H, each CCH_3), 1.67 (br. s, 1H, CN*H*), 1.54 (m, 4H, CCH_2CH_2), 1.38, 1.35 (each s, 15H, C(CH_3)$_3$ and C(CH_3)$_2$).

Synthesis of 23: Compound **19** (469 mg, 1.3 mmol) and NHS (298 mg, 2.59 mmol) was dissolved in tetrahydrofuran (15 mL) and then DIC (405 μL, 2.59 mmol) was added. The mixture solution was stirred at room temperature for 3 h. Later the above solution was added dropwise to a well stirred water solution (3mL) of compound **22** (300 mg, 0.26 mmol) which the pH was adjusted to 8.5 by sodium carbonate. The mixture was stirred at room temperature for 15 h. The solvent was evaporated under reduced pressure. The residue was washed by water, filtered, and purified by flash chromatography on silica gel to produce **23** as a white powder (205 mg, 52.4 %). ^{1}H NMR (400 MHz, DMSO-d_6) δ: 8.38 (d, 1H, N*H*), 8.23 (m, 3H, N*H* and adenine H overlapping), 8.12 (m, 4H, N*H* and adenine H overlapping), 7.79 (d, 1H, ArH), 7.73 (m, 2H, ArH), 7.76 (s, 1H, ArH), 7.42 (m, 2H, ArH), 7.33 (d, 1H, ArH), 7.20 (m, 5H, ArH), 6.70, 6.42 (each br. s, 2H, N*H*CN*H*), 5.92 (d, 1H, H-1), 5.37 (t, 1H, H-4), 5.02 (t, 1H, H-3), 4.90 (t, 1H, H-2), 4.61 (m, 2H, C*H*CH$_2$CO and C*H*CH$_2$C overlapping), 4.49 (m, 1H, C*H*CH$_2$C), 4.36 (d, 1H, H-5), 4.30 (m, 1H, C*H*CH$_2$CH$_2$), 4.20 (t, 2H, NHCH$_2$CH_2N), 3.73 (br. s, 2H, NHCH_2CO), 3.41 (m, 2H, NHCH_2CH$_2$N), 3.03 (m, 4H, C*H*CH$_2$C), 2.89 (m, 4H, CCH_2C and CHCH_2CO overlapping), 2.59 (m, 2H, CH$_2$CH_2NH), 2.47, 2.40, 1.84 (each s, each 3H, each CCH_3), 2.05, 2.01, 1.99, 1.97 (each s, each 3H, each

COCH_3), 1.67 (br. s, 1H, CN*H*), 1.53 (m, 4H, CCH_2CH_2), 1.37, 1.34 (each s, 15H, C(CH_3)$_3$ and C(CH_3)$_2$). LCMS (ESI) (m/z): $C_{72}H_{90}N_{14}O_{20}S$, calcd 1502.62; found 1501.86 $[M-1]^-$, 1503.81 $[M+1]^+$.

Synthesis of 24: Compound **23** (205 mg, 0.14 mmol) was dissolved in 10 mL TFA/H_2O (95: 5) and stirred at room temperature for 1 h. TFA was removed by air blowing, and diethyl ether was added. The precipitate formed was filtered off under reduced pressure to obtain a light yellow crude product **24** which was used directly for synthesis of **3**.

Synthesis of 3: The crude product **24** was suspended in a mixture of MeOH/Et_3N/H_2O (4:1:5, 14 mL) and stirred at room temperature for 2 h. The solution was concentrated under reduced pressure and the residue was purified by HPLC. Freeze-drying gave a white powder **3** (42.9 mg, 25.6 %). ^{1}H NMR (400 MHz, DMSO-d_6) δ: 8.22 (m, 5H, N*H* and adenine H overlapping), 8.08 (m, 3H, N*H* and adenine H overlapping), 7.79 (m, 4H, ArH), 7.47 (m, 2H, ArH), 7.32 (d, 1H, ArH), 7.26 (m, 5H, ArH), 4.90 (s, 1H, H-1), 4.46 (m, 3H, C*H*CH_2CO, H-2, and C*H*CH_2C overlapping), 4.33 (m, 2H, C*H*CH_2C and C*H*CH_2CH_2), 4.06 (d, 1H, H-5), 3.75 (m, 2H, NHCH_2CO), 3.63 (m, 4H, NHCH_2CH_2N), 3.48 (t, 1H, H-4), 3.31 (m, 1H, H-3), 3.22 (m, 4H, O*H*), 3.09 (m, 4H, C*H*CH_2C), 2.87 (m, 2H, CHCH_2CO), 2.63 (m, 2H, CH_2CH_2NH), 1.76 (br. s, 1H, CN*H*), 1.52 (m, 4H, CCH_2CH_2). LCMS (ESI) (m/z): $C_{47}H_{58}N_{14}O_{13}$, calcd 1026.43; found 1025.33 $[M-1]^-$, 1027.41 $[M+1]^+$.

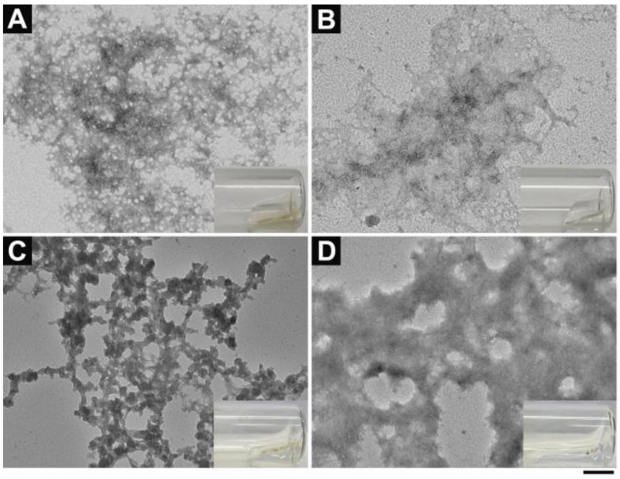

Figure S1: TEM images of solutions of (A) **1**, (B) **2**, (C) **5**, (D) **6**. All compounds were dissolved in DI water at the concentration of 3.0 wt% and pH 4.0. Inserts are the corresponding optical images. Scale bar = 100 nm.

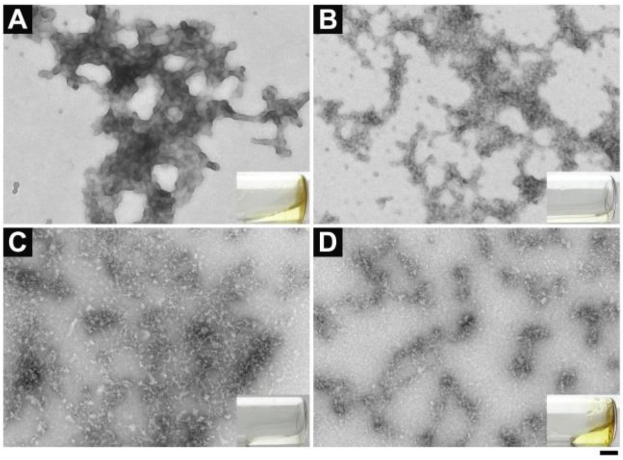

Figure S2: TEM images of solutions of (A) **3** (pH = 6.2, 3 wt %), (B) **4** (pH = 6.8, 3 wt %), (C) **7** (pH = 5.0, 3 wt %), (D) **8** (pH = 5.4, 3 wt %). Inserts are the corresponding optical images. Scale bar = 100 nm.

References

1. Pandey, M. D.; Mishra, A. K.; Chandrasekhar, V.; Verma, S. *Inorg. Chem.,* **2010**, *49*, 2020-2022.

2. Elidrisi, I.; Negin, S.; Bhatt, P. V.; Govender, T.; Kruger, H. G.; Gokel, G. W.; Maguire, G. E. M. *Org. Biomol. Chem.,* **2011**, *9*, 4498-4506.

3. Das, B. K.; Shibata, N.; Takeuchi, Y. *J. Chem. Soc., Perkin Trans. 1,* **2002**, 197-206.

4. Tosin, M.; Murphy, P. V. *Org. Lett.,* **2002**, *4*, 3675-3678.

5. Cagnoni, A. J.; Varela, O.; Gouin, S. G.; Kovensky, J.; Uhrig, M. L. *J. Org. Chem.,* **2011**, *76*, 3064-3077.

6. Chan, W. C.; white, P. D. In *Fmoc Solid Phase Peptide Synthesis: A Practical Approach*; Oxford University Press Inc.: New York, 2000, p 41-73.
